# Retinal nerve fibre layer thickness measurements in childhood glaucoma: the role of scanning laser polarimetry and optical coherence tomography

**DOI:** 10.1007/s00417-021-05276-z

**Published:** 2021-06-26

**Authors:** Mael Lever, Christian Halfwassen, Jan Darius Unterlauft, Nikolaos E. Bechrakis, Anke Manthey, Michael R. R. Böhm

**Affiliations:** 1 Department of Ophthalmology, University Hospital Essen, Hufelandstr. 55, 45147 Essen, Germany; 2 Achim Wessing Institute for Imaging in Ophthalmology, University Hospital Essen, Essen, Germany; 3 Department of Ophthalmology, University Hospital Leipzig, Leipzig, Germany

**Keywords:** Childhood glaucoma, Primary congenital glaucoma, Retinal nerve fibre layer, Optical coherence tomography, Scanning laser polarimetry, Imaging

## Abstract

**Purpose:**

A central diagnostic tool in adult glaucoma is the peripapillary retinal nerve fibre layer (pRNFL) thickness. It can be assessed by scanning laser polarimetry (SLP) or optical coherence tomography (OCT). However, studies investigating the relevance of pRNFL measurements in children are rare. This study aims to compare the glaucoma diagnosing ability of SLP and OCT pRNFL thickness measurements in a paediatric population.

**Methods:**

This retrospective study included 105 children (glaucoma: 22 (21.0%); healthy glaucoma suspects: 83 (79.0%)) aged 4–18 years, examined with SLP (GDxPro/ECC, Carl Zeiss Meditec) and spectral-domain OCT (SPECTRALIS®, Heidelberg Engineering). The thickness of pRNFL sectors was compared between diseased and healthy participants. Areas under the receiver-operating characteristic curves (AUC) and logistic regression results were used to compare the glaucoma discriminative capacity between SLP and OCT measurements.

**Results:**

Using OCT, pRNFL thickness was decreased in the superior, nasal, and inferior quadrants of glaucoma patients compared to healthy controls (*P* < 0.001, each). With SLP, such a difference was only observed in the inferior quadrant (*P* = 0.011). A correlation between glaucoma diagnosis and OCT-measured pRNFL thickness was found in all quadrants (*P* < 0.001) other than the temporal. With SLP, a correlation was found for the total average thickness (*P* = 0.037) and inferior quadrant (*P* = 0.0019). Finally, the AUCs of OCT measurements were markedly higher than those of SLP (e.g., inferior quadrant: OCT 0.83, SLP 0.68).

**Conclusion:**

pRNFL thickness measurements using both OCT and SLP, correlate notably with the presence of glaucoma. In general, the diagnostic performance of pRNFL thickness measurements seems higher for OCT than for SLP. Thus, pRNFL thickness measurements could provide important information, complementing conventional clinical and functional parameters in the diagnostic process of paediatric glaucoma. 
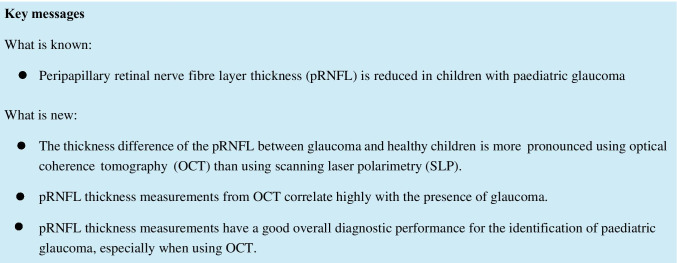

## Introduction

Childhood glaucoma is defined as a group of diseases characterised by a progressive damage to retinal ganglion cells, the optic nerve, and other ocular structures due to elevated intraocular pressure (IOP) [1]. Although childhood glaucoma is considered a rare disease and the prevalence of its various aetiologies varies widely around the world, it is likely to be the cause of approximately 5% of blindness in children [2]. The diagnosis of childhood glaucoma is based on elevated IOP, morphologic changes of the optic nerve head (ONH), corneal abnormalities (Haab striae, enlarged diameter), progressive myopia or axial length, and glaucomatous visual field (VF) defects [3]. Regardless of aetiology, paediatric glaucoma often poses a diagnostic challenge. One reason is the difficulty in performing slit lamp or fundoscopic evaluation in young patients. Also, their limited ability to cooperate and their short attention span further impedes the reliability of functional tests (e.g., perimetry), and IOP measurements [4]. Finally, most of the diagnostic equipment is designed for adult rather than paediatric patients [5] and normative databases with healthy and pathologic measurements of children of various ages are lacking.

In addition to the previously mentioned criteria, methods have been developed to objectively quantify retinal glaucomatous damage, in particular the loss of retinal ganglion cells (RGC). The loss of RGCs manifests itself in a thinning of the retinal nerve fibre layer (RNFL) around the ONH and in the macular region [6]. Precise measurement of the peripapillary RNFL (pRNFL) has become a key parameter of glaucoma diagnostic and follow-up in adults [7].

pRNFL thickness can be assessed using two main methods: scanning laser polarimetry (SLP) [8] and optical coherence tomography (OCT) [9]. The determination of pRNFL thickness using SLP is based on the measurement of light retardation passing through the birefringent neuronal axons mainly located in the RNFL [10]. Introduction of the first SLP instruments dates back to the early 1990s and technical improvements were focused on better compensation of the cornea's and the lens’ own birefringence (enhanced corneal compensation, ECC) as well as optimization of software-based glaucoma identifying parameters such as the TSNIT score (temporal-superior-nasal-inferior-temporal) and nerve fibre indicator (NFI) [11]. Despite not being commercially available any more, the high value of SLP for glaucoma diagnostic and follow-up is well documented [12] and still widely used [13]. OCT measurements are based on the interferometry of low-coherence light from a diode or laser reflected from the retinal layers compared to a reference mirror. The result can be visualised as a cross sectional, 3-dimensional measurement of the retina and ONH [14]. With the technical improvements to the instruments since the early 1990s, OCT measurements became faster and the axial resolution was improved [15]. In glaucoma, OCT can detect a decrease in RNFL thickness [6], which correlates with a decline of visual function (i.e. VF defects) [16]. Furthermore, the high intervisit reproducibility of OCT measurements legitimates its utility for the follow-up of glaucoma patients [17, 18].

pRNFL measurements using SLP and OCT proved to be equally precise in adults (reviewed in a Cochrane report by Michelessi et al. [19]). The value of OCT as a diagnostic method in childhood glaucoma has been investigated in small cohorts [17, 18, 20]; however, comparisons of both SLP and OCT in a paediatric context are scarce [21]. Beyond that, the latest technical improvements of OCT and SLP instruments allowing faster, more precise examinations facilitate their usage in children and improve the reliability of their results in paediatric patients. Therefore, a detailed comparison of both techniques’ current version in this population seems highly relevant.

The aim of this study was to examine the differences in pRNFL thickness between paediatric glaucoma patients and healthy children using both SLP (GDx Pro with ECC) and spectral domain OCT (SD-OCT), in order to compare their respective diagnostic potential in the context of childhood glaucoma.

## Materials and methods

### Study design

Retrospective chart analysis of glaucoma patients and healthy glaucoma suspects ≤ 18 years of age who received a pRNFL measurement with both OCT and SLP in our Ophthalmology Department between 2011 and 2018. The study was conducted in accordance with the Tenets of the 1964 Declaration of Helsinki and was approved by the ethics committee of the University Hospital Essen, Germany (approval number: 16–7114-BO). Exclusion criteria of the study were systemic diseases, prematurity, any non-glaucomatous condition apart from strabismus (especially optic atrophy, papilledema, amblyopia), missing data regarding IOP, best-corrected visual acuity (BCVA), anterior segment examination, and/ or fundoscopy, e.g., due to a lack of participation. Control participants were healthy children referred to our ophthalmology department due to glaucoma suspicion because of a suspect ONH morphology or borderline IOP in a screening examination, but for whom glaucoma was ruled out after follow-up examinations.

### Glaucoma diagnosis

Childhood glaucoma was evaluated based on the ninth Consensus Report of the World Glaucoma Association [22] and the recommendation of the Childhood glaucoma Research Network [3]. Diagnosis was confirmed in the presence of at least 2 of the following criteria: elevated IOP measurements, ONH morphology (cupping, focal rim loss), corneal changes typical of glaucoma (Haab striae), enlarged corneal diameter, increasing axial length or myopia. If necessary, these examinations were performed in general anaesthesia.

Measurements of the pRNFL were performed using SLP and OCT on the same day along with a comprehensive ophthalmic examination, including a review of the past medical history and current therapy, determination of BCVA, slit-lamp examination of anterior segment, fundoscopy (including evaluation of ONH linear cup-to-disc ratio, CDR), IOP-measurement (Goldmann applanation tonometer or Perkins Mk2 Hand-held applanation tonometer, Haag-Streit, Bern, Switzerland), and if possible stereoscopic ONH photography and visual field examination using 30–2 static automated perimetry (Twinfield 2, OCULUS Optikgeräte, Wetzlar, Germany). Data of the right eye was selected for further analysis whenever possible.

### SLP measurements

For pRNFL thickness assessment by laser-scanning polarimetry (GDx Pro/ECC, Carl Zeiss Meditec, Oberkochen, Germany), a fixed measurement band with an inner circle diameter of 2.4 mm and outer diameter of 3.2 mm is centred to the ONH after determination of anterior segment birefringence. The device measures 256 retardation values around the peripapillary band and translates the results into RNFL thickness. The typical scan score (TSS) was 80 or higher and the image quality score was ≥ 8. The used software version 1.1.1.14 generally provides a value for the superior and inferior average thickness and TSNIT score (total average RNFL thickness). In our paediatric population, the common nerve fibre indicator (NFI) was not available due to the lack of a paediatric normative database. The RNFL thickness values for the superior (225°–315°), nasal (315°–45°), inferior (45°–135°), and temporal (135°–225°) quadrants of the peripapillary band were exported from the extended examination results’ export file.

### OCT measurements

pRNFL measurements by Spectral domain optical coherence tomography (SD-OCT) were performed using a SPECTRALIS® SD-OCT (Heidelberg Engineering, Heidelberg, Germany). Therefore, a circular scan of 3.5 mm diameter centred to the ONH is projected and a full RNFL circle containing 768 A-scans is acquired. The manufacturer’s software (version 1.7.1.0) divides the obtained disc into four 90° quadrants (superior S, nasal N, inferior I, and temporal T) and averages them as total average thickness. Additionally, the superior and inferior quadrants are both divided in 45° sectors (temporal superior TS, nasal superior NS, nasal inferior NI, and temporal inferior TI). Prior to processing the thickness measurements, each scans’ quality was assessed using the OSCAR-IB consensus criteria [23]. In case of insufficient quality, scans were excluded. If necessary, layer alignment was corrected manually. The software measures image quality using a score ranging from 0 (poor quality) to 40 (excellent quality); all images included for further analysis showed an image quality score ≥ 20.

### Statistical analysis

The numeric data was gathered in Microsoft Excel (Microsoft, Redmond, WA, USA). Normality was examined using the D’Agostino and Pearson normality test. Mean values were compared applying Student’s two-sided unpaired *t*-test, or Mann–Whitney *U* test, when appropriate. Correlation between parameters were evaluated calculating Pearson or Spearman correlation factors when appropriate; univariate logistic regression analyses were performed using Prism 9 for Mac (GraphPad, La Jolla, CA, USA), reporting coefficients, the standard error, Tjur’s pseudo *R*^2^, and *p*-value of the log-likelihood ratio. In the results section of this article, numeric results are presented as “mean ± standard deviation” or as “median (confidence level of the median (CL))” when appropriate. In addition, sensitivity and specificity were calculated, receiver-operator characteristic (ROC) curves were plotted and the area under the ROC curve (AUC) was measured for both SLP and OCT diagnostic techniques. In general, statistical significance was assumed for *p* < 0.05.

## Results

### Patients characteristics

**Fig. 1:**
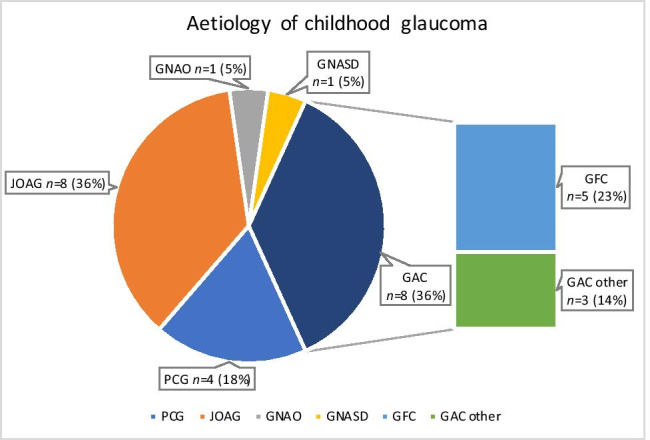
Distribution of glaucoma aetiologies. The diagram shows the relative distribution of glaucoma aetiologies in the presented cohort of paediatric glaucoma patients (*n* = 22) using the classification recommended by the Childhood Glaucoma Research Network (https://wga.one/wga/consensus-9/). PCG, primary congenital glaucoma; JOAG, juvenile primary open-angle glaucoma; GFC, glaucoma following cataract surgery; GNASD, glaucoma associated with non-acquired systemic disease or syndrome; GNAO, glaucoma associated with non-acquired ocular anomalies; GAC, glaucoma associated with acquired conditions

SLP and OCT results of 105 children aged 4.1 to 18.2 (mean 11.6 ± 3.1) years were analysed. 43.8% (*n* = 46) were 10 years old or younger at the time of examination. In the study population, female gender was slightly underrepresented with 44.8% (*n* = 47); 22 children (21%) were previously diagnosed with glaucoma, 10 of them (45.5%) were younger than 10 years. With 8 participants (36.4%) each, the main cause of glaucoma in our cohort was juvenile open-angle glaucoma and “glaucoma associated with acquired conditions” (including 5 patients (22.7%) with glaucoma following cataract surgery); 4 patients (18.2%) had a primary congenital glaucoma. The detailed distribution of aetiologies of glaucoma is represented in Fig. 1. The median BCVA was 0.0 LogMAR (CL control 95.2%; CL glaucoma 98.3%). The mean IOP was 14.0 ± 2.5 mm Hg for glaucoma patients and 14.9 ± 3.4 mm Hg in the control group (*p* = 0.0002). Antiglaucomatous therapy in the glaucoma group consisted of a median of 3 (CL 98.3%) topical and/or systemic antiglaucomatous agents. The mean deviation of static automated perimetry was − 3.7 ± 4.2 dB for glaucoma patients and − 3.1 ± 4.5 dB in controls (*p* = 0.30). Further epidemiologic data are shown in Table 1.

**Table 1:** Epidemiologic and general ophthalmologic characteristics of patients

*Patients*	*(n)*	*105*
*Gender*	male:female % (n)	55.2:44.8% (58:47)
*Diagnosis*	glaucoma:controls % (n)	21.0:79.0% (22:83)
*Eye*	right:left % (n)	92.4:7.6% (97:8)
*Age*	mean ± SD (y)	11.6 ± 3.1
	glaucoma	11.7 ± 3.7
	controls	11.5 ± 3.8
*BCVA*	median (95% CL) (LogMAR)	0.0 (96.9%)
*IOP*	mean ± SD (mm Hg)	14.7 ± 3.3
	glaucoma	14.0 ± 2.5
	controls	14.9 ± 3.4
*SAP (mean deviation)*	mean ± SD (dB)	− 3.2 ± 4.4
	glaucoma	− 3.7 ± 4.2
	controls	− 3.1 ± 4.5
*Follow-up time*	mean ± SD (m)	16.7 ± 16.4

The table shows the main characteristics of patients included in the present study. *BCVA*, best-corrected visual acuity; *IOP*, intraocular pressure; *SD*, standard deviation; *CL*, confidence level of the median; *y*, years; *m*, months; *mm Hg*, millimetre of mercury; *SAP*, static automated perimetry; *dB*, decibel

### Measurements of the pRNFL thickness with OCT and SLP

First, both instruments’ pRNFL thickness measurements of glaucoma patients and healthy controls were compared. As expected, OCT results revealed a significantly thinner pRNFL in glaucoma patients compared to controls (Table 2). In particular, this was found for total pRNFL thickness as well as in the superior, nasal, and inferior sectors but not in the temporal (e.g., total average thickness: glaucoma: 79.9 ± 19.4 µm; controls: 98.3 ± 7.7 µm; *p* < 0.001). Using SLP, no significant difference of pRNFL thickness was visible between both groups (e.g., total average thickness: glaucoma: 49.7 ± 7.9 µm; controls: 52.8 ± 6.0 µm; *p* = 0.30) except for the inferior quadrant (glaucoma: 60.8 ± 12.6; controls: 67.8 ± 7.6; *p* = 0.011).

**Table 2:** pRNFL thickness is reduced in glaucoma patients compared to healthy individuals

	*pRNFL thickness*		
	Glaucoma mean ± SD (µm)	Controls mean ± SD (µm)	p-value
*SLP*			
Total average	49.7 ± 7.9	52.8 ± 6.0	0.30
Superior	58.8 ± 12.3	63.0 ± 8.0	0.14
Nasal	33.2 ± 8.5	33.6 ± 10.2	0.52
Inferior	60.8 ± 12.6	67.8 ± 7.6	**0.011**
Temporal	24.7 ± 13.0	19.9 ± 9.7	0.53
*OCT*			
Total average	79.9 ± 19.4	98.3 ± 7.7	** < 0.001**
Superior	90.8 ± 33.2	121.7 ± 14.7	** < 0.001**
Nasal	58.3 ± 16.7	75.7 ± 13.0	** < 0.001**
Inferior	93.0 ± 31.8	126.2 ± 13.6	** < 0.001**
Temporal	67.2 ± 18.5	70.2 ± 9.3	0.59

The table shows the mean thickness of peripapillary retinal nerve fibre layer (pRNFL) thickness measured by scanning laser polarimetry (SLP) and optical coherence tomography (OCT). *SD*, standard deviation. Statistically significant results when *p* < 0.05 (bold and underlined)

In general, the pRNFL thickness provided by SLP was lower compared to measurements from OCT. To further investigate this observation, we assessed the correlation between both methods’ measurements. In the glaucoma group, a strong correlation between SLP and OCT appeared for the total average pRNFL thickness (correlation factor *r* 0.62; 95% CI 0.26–0.82; *p* = 0.001), the superior (r, 0.60; 95% CI: 0.24–0.82; *p* = 0.0016), and inferior quadrants (*r*, 0.73; 95% CI, 0.44–0.88; *p* < 0.001). In contrast, the correlation between OCT and SLP results was generally lower in the control group and only statistically significant for the total average pRNFL thickness (*r*, 0.20; 95% CI, 0.023–0.40; *p* = 0.035) and nasal quadrant thickness (*r*, 0.32; 95% CI, 0.10–0.50; *p* = 0.0017) (Table 3).

**Table 3:** Correlation of pRNFL thickness measurements from SLP and OCT

	*r*	95% CI	*p*-value
*Glaucoma*			
Total average	0.62	0.26–0.82	**0.001**
Superior	0.60	0.24–0.82	**0.0016**
Nasal	0.058	− 0.38 to 0.48	0.40
Inferior	0.73	0.44–0.88	** < 0.001**
Temporal	0.13	− 0.32 to 0.53	0.28
*Controls*			
Total average	0.20	− 0.023 to 0.40	**0.035**
Superior	0.15	− 0.071 to 0.36	0.083
Nasal	0.32	0.10–0.50	**0.0017**
Inferior	0.093	− 0.13 to 0.30	0.20
Temporal	0.045	− 0.18 to 0.26	0.34

This table shows the correlation between peripapillary retinal nerve fibre layer (pRNFL) thickness measurements from scanning laser polarimetry (SLP) and optical coherence tomography (OCT). Results in cases and controls are presented separately.*r*, Pearson or Spearman correlation factor, when appropriate; *CI*, confidence interval; statistically significant results when *p* < 0.05 (bold and underlined)

### Correlation between glaucoma diagnosis and pRNFL thickness

To evaluate the potential correlation between pRNFL thickness and the presence of glaucoma, Spearman correlation factors were calculated. For SLP measurements, a correlation was found in the inferior quadrant (*r*, 0.25; 95% CI, 0.052–0.42; *p* = 0.0055). Furthermore, logistic regression analyses revealed a correlation for SLP measurements for the total, inferior and temporal quadrants (e.g., total average pRNFL thickness: coefficient, − 0.084; odds ratio, 0.92; *p* = 0.037; Table 4). However, the positive correlation coefficient for the temporal quadrant (coefficient, 0.061; odds ratio, 1.06; *p* = 0.024) suggests a correlation of higher pRNFL thickness with the presence of glaucoma. This contradicts common clinical knowledge and should therefore be interpreted carefully. For OCT-measured pRNFL thickness, a correlation was found for the total average pRNFL thickness (*r*, 0.45; 95% CI, 0.28–0.59; *p* < 0.001), the superior (*r*, 0.37; 95% CI, 0.19–0.53; *P* < 0.001), nasal (*r*, 0.41; 95% CI, 0.23–0.56; *P* < 0.001), and inferior quadrants (*r*, 0.47; 95% CI, 0.30–0.61; *p* < 0.001; Table 4). This notable correlation was further undermined by the logistic regression results (e.g., total average thickness: coefficient, − 0.14; *R*^2^, 0.39; *p* < 0.001) presented in Table 4.

**Table 4:** Correlation between pRNFL thickness and glaucoma diagnosis

	Correlation			Logistic regression			
	r	95% CI	p value	Coefficient	Standard error	R^2^	p value
SLP							
Total	0.10	− 0.096 to 0.29	0.15	− 0.084	0.042	0.050	0.037
Superior	0.13	− 0.066 to 0.32	0.088	*N.N*	*N.N*	0.044	*N.N*
Nasal	0.063	− 0.14 to 0.26	0.26	*N.N*	*N.N*	< 0.001	*N.N*
Inferior	0.25	0.052–0.42	0.0055	− 0.082	0.029	0.11	0.0019
Temporal	− 0.062	− 0.26 to 0.14	0.27	0.061	0.027	0.064	0.024
OCT							
Total	0.45	0.28–0.59	< 0.001	− 0.14	0.036	0.39	< 0.001
Superior	0.37	0.19–0.53	< 0.001	− 0.066	0.016	0.34	< 0.001
Nasal	0.41	0.23–0.56	< 0.001	− 0.095	0.024	0.27	< 0.001
Inferior	0.47	0.30–0.61	< 0.001	− 0.077	0.019	0.38	< 0.001
Temporal	0.053	− 0.15 to 0.25	0.30	*N.N*	*N.N*	0.017	*N.N*

This table shows the correlation between pRNFL thickness and glaucoma diagnosis illustrated by Spearman correlation calculation and results from logistic regression of the peripapillary retinal nerve fibre layer (pRNFL) thickness measured by scanning laser polarimetry (SLP) and optical coherence tomography (OCT). *r*, Spearman correlation factor; *CI*, confidence interval; *R*^2^, Tjur’s pseudo *R*^2^ factor; statistically significant results when *p* < 0.05 (bold and underlined)

### Glaucoma diagnostic performance of SLP and OCT

**Fig. 2:**
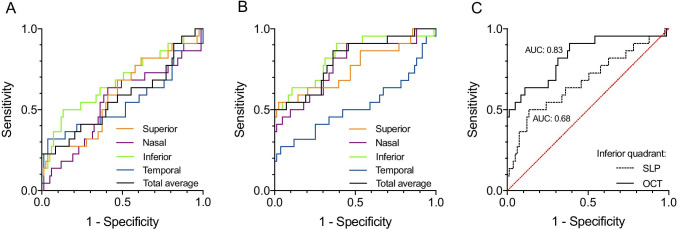
ROC curves for SLP and OCT measurements of pRNFL thickness. Figure 2A shows the receiver-operator characteristic (ROC) curves for total peripapillary retinal nerve fibre layer (pRNFL) thickness measurements as well as thickness measurements in the superior, nasal, inferior and temporal quadrants with scanning laser polarimetry (SLP). Figure 2B show ROC for these parameters measured by optical coherence tomography (OCT). Figure 2C compares the best performer SLP and OCT parameter (inferior quadrant, both) and presents their respective area under the ROC curve (AUC)

To evaluate the ability of both diagnostic methods to differentiate between healthy and glaucoma participants in the present study population, the ROC curves for the pRNFL measurements by SLP and OCT were plotted (Fig. 2) and the area under the ROC (AUC) was analysed (Table 5). With SLP, the AUC was statistically significant only in the inferior quadrant (AUC, 0.68; 95% CI, 0.54–0.81; *p* = 0.011). For other quadrants, AUC ranged from 0.54 nasally and temporally to 0.59 (95% CI, 0.46–0.73; *p* = 0.17) in the superior quadrant (Fig. 2A). In contrast, OCT results returned a significant AUC for all peripapillary segments except for the temporal. In particular, the AUCs were higher for OCT measurements than for SLP, ranging from 0.72 (95% CI, 0.58–0.85; *p* = 0.0018) in the nasal superior sector to 0.83 (95% CI, 0.73–0.94; *p* < 0.001) in the inferior quadrant (Fig. 2B). The thickness of the inferior quadrant was the best-performing parameter using both SLP and OCT. Detailed results are presented in Fig. 2. More detailed data (e.g., AUC analysis) for all quadrants and sectors are displayed in Table 5.

**Table 5:** Area under the ROC curve and sensitivity of OCT than for SLP measurements for the presence of glaucoma

				Sensitivity		
AUC		95% CI	p-value	Specificity ≥ 80%	Specificity ≥ 90%	Specificity ≥ 95%
SLP						
Total average	0.57	0.42–0.72	0.30	32%	27%	23%
Superior	0.59	0.46–0.73	0.17	27%	27%	23%
Nasal	0.54	0.40–0.69	0.52	18%	14%	4.5%
Inferior	0.68	0.54–0.81	**0.011**	50%	36%	23%
Temporal	0.54	0.38–0.70	0.53	36%	32%	32%
OCT						
Total average	0.82	0.71–0.92	** < 0.001**	59%	55%	50%
Superior	0.76	0.63–0.89	** < 0.001**	59%	55%	55%
Temp. sup	0.78	0.65–0.91	** < 0.001**	64%	64%	50%
Nas. sup	0.72	0.58–0.85	**0.0018**	59%	55%	55%
Nasal	0.79	0.67–0.90	** < 0.001**	55%	50%	50%
Inferior	0.83	0.73–0.94	** < 0.001**	64%	59%	55%
Nas. Inf	0.81	0.70–0.92	** < 0.001**	64%	50%	45%
Temp. inf	0.78	0.65–0.91	** < 0.001**	55%	55%	55%
Temporal	0.54	0.38–0.70	0.59	55%	27%	27%

Values of the area under the receiver-operator characteristic curve (AUC) and its 95% confidence interval (95% CI) as well as sensitivity values at fixed specificity of 80%, 90%, and 95% are tabularized for peripapillary retinal nerve fibre layer thickness measurements with scanning laser polarimetry (SLP) and optical coherence tomography (OCT). The AUC is statistically different from 0.5 (statistically significant) when *p* < 0.05 (bold and underlined

Finally, these analyses allow a direct comparison of sensitivity and specificity for both diagnostic methods. At fixed specificities of 80%, 90%, and 95% using SLP, sensitivity was highest for the inferior pRNFL thickness (sensitivity 23–50%) but was otherwise low. Regarding OCT measurements, sensitivity at these fixed specificities was generally higher than for SLP measurements. Here, the measurements of the temporal superior sector and of the inferior quadrant yielded the best results (e.g., 64% at 80% specificity, Table 5).

## Discussion

The present study addresses the role of peripapillary RNFL thickness measurements for diagnosing childhood glaucoma comparing two widely available methods, SLP and SD-OCT. The principal findings of the study are as follows:

1. In general, pRNFL thickness measurements are lower in childhood glaucoma patients than in healthy controls. The pRNFL thickness difference between glaucoma and control participants is more pronounced when using OCT than using SLP.
2. The reduced pRNFL thickness measured using OCT in children with glaucoma correlates highly with the presence of the disease.
3. The diagnostic performance of OCT assessed pRNFL thickness is good and is markedly higher than that of SLP.

Imaging techniques like SLP and OCT provide precise objective information about retinal morphology (e.g., the pRNFL). In glaucoma diagnostic, they constitute an important addition to more subjective and/or functional methods such as perimetry or the fundoscopic evaluation of the ONH morphology [24]. Although both SLP and OCT allow measurements of pRNFL thickness, they make use of different methods. While SLP measures the light retardation of the nerve fibres in the RNFL [10], OCT compares the light backscatter from the RNFL to the light reflected from a reference mirror [14]. These technical differences encumber a comparison of both diagnostic tools, which is further exacerbated by the fact that several instruments iterations and multiple software versions are actively used [25, 26].

Peripapillary RNFL thickness is considered a crucial parameter for glaucoma diagnosis and monitoring in adults [27]. On the contrary, in paediatric glaucoma, its role has as yet not been conclusively understood and the diagnostic reliability of both SLP and OCT methods is still uncertain. Furthermore, normative data of pRNFL thickness in healthy as well as glaucoma-affected infants of various age groups are lacking. In this paper, we present pRNFL thickness data from SLP (GDxPro/ ECC) and SD-OCT of 22 glaucoma patients and 83 healthy control individuals aged 4 to 18 years. In this cohort, glaucoma aetiologies are similarly distributed as in previous population reports [1] except for an overrepresentation of juvenile open-angle glaucoma and an underrepresentation of primary congenital glaucoma (PCG). This can be explained by the exclusion criteria used in this study: high image quality was obligatory for the study analyses; in PCG, the frequent significant media opacity that leads to poor image quality in SLP and OCT, resulted in an exclusion from the study.

In the present paediatric population, pRNFL thickness measurements by SLP are significantly reduced in glaucoma patients, but solely in the inferior quadrant. In contrast, OCT measurements are significantly reduced in glaucoma patients in all quadrants except for the temporal. Also, pRNFL thickness was consistently lower when measured by SLP rather than OCT, which is in line with results in adults [28]. Also, the present results show a correlation of several pRNFL measurements between both SLP and OCT methods in glaucoma patients (e.g., total average thickness, pRNFL thickness in the superior and inferior quadrants). This is consistent with previous studies in children [21] and adults [25], which describe a correlation between the TSNIT score of SLP and the total average pRNFL thickness of OCT as well as between the superior average and inferior average pRNFL thickness of SLP and superior and inferior quadrant thickness of OCT, respectively. However, the presented results show that SLP and OCT measurements rarely correlate in healthy children. The reason for this difference between diseased and healthy children is unclear. One explanation could be that, in controls, the standard deviation of the total average pRNFL thickness is high (e.g., up to 10 µm in SLP and up to 14 µm in OCT), due to general interindividual—possibly growth-related—variability. In addition, changes of physical properties of each anatomical structure (e.g., cornea, lens, vitreous, posterior hyaloid membrane) like transparency, reflectivity, and birefringence may impact SLP and OCT measurements differently [29]. While these issues also affect measurements in glaucoma participants, it may be that the glaucoma-induced general decrease of pRNFL thickness outweighs this interindividual variability. Additionally, further study limitations need to be considered. The wide interindividual variation of measurements between subjects aged 4 to 18 cannot be compensated by the relatively small number of included glaucoma cases (*n* = 22). In addition, some interfering factors affecting SLP and OCT measurements (e.g., glaucoma aetiology, ONH size, refractive error) and results classification [26] could not be examined separately. Finally, the average disease severity evaluated by perimetry was low (mean deviation of − 4.1 ± 6.5 dB in the glaucoma group); thus, our population only represents a small portion of disease severity. Regarding the comparability of the present results, another particularity here is that to better compare temporal and nasal results of SLP with the quadrant results from OCT, SLP quadrant measurements were calculated manually using the internal pRNFL results. Consequently, the presented SLP superior and inferior quadrant results are not comparable to the values of “superior average” and “inferior average” used in the previous literature (which correspond to pRNFL thickness in the superior and inferior semicircle, respectively). In summary, even though our data show some correlation between SLP and OCT measurements, as in adults [28], results of both methods in children are not equivalent nor interchangeable.

Using logistic regression analysis, the association of the pRNFL thickness and glaucoma diagnosis was examined. The results show a strong correlation of OCT measurements with the presence of glaucoma (e.g., total average thickness, pRNFL thickness in all quadrants but the temporal), whereas such a correlation is missing when using SLP except for total average pRNFL thickness and measurements of the inferior quadrant. Regarding OCT, these observations are in line with studies conducted with adult participants, where pRNFL thickness is reduced in glaucoma patients compared to healthy controls, with the greatest difference in the infero- and superotemporal sectors [30, 31]. Similarly, studies conducted on primary congenital glaucoma patients report differences between glaucoma patients and controls in all peripapillary sectors [26, 32, 33]. Together, these findings suggest that using the parameters available for children, OCT could be more valuable for diagnosing childhood glaucoma than SLP.

To further examine this hypothesis, area under the ROC curves, sensitivity, and specificity data were assed for both methods. The presented analysis confirms previous observations as the AUC of OCT results is consistently greater than those of SLP. Apart from this, statistical significance is reached for all OCT parameters except for the temporal quadrant pRNFL thickness, as opposed to SLP measurements, which are statistically significant only in the inferior quadrant. Observations of sensitivity and specificity confirm these findings: at fixed specificities of 80%, 90%, and 95%, sensitivity was markedly lower for SLP measurements compared to OCT. Previously, in adults, a good glaucoma diagnostic performance (as by AUC calculation) was observed for pRNFL measurements by SLP [8, 34] as well as OCT [34] and both methods have been reviewed as equivalent in a Cochrane report [19] as well as in a recent large cohort study [13] when considering best performing parameters. In particular, SLP parameters with the highest AUC are the nerve fibre indicator (NFI) and the TNSIT score, which are both calculated values without an OCT surrogate. Similarly, a study by Zareii et al. [21] including 24 juvenile open-angle glaucoma patients and 24 healthy controls also reported high AUC for OCT parameters and even higher values for SLP’s NFI. However, the patients in this study were aged 11 to 40 years with a mean of 25 ± 8 years, which is not comparable with the present cohort. NFI represents an age-matched probability of glaucoma relying on a database of 540 healthy individuals and 262 glaucoma patients aged 18 to 82 years (Michael Sinai, PhD, Laser Diagnostic Technologies Inc., written communication, March 2003) and has a high discriminative value for glaucoma in adults [12]. Lacking paediatric reference data, NFI is not available for the young patients in our study. Another inconvenience of using NFI in glaucoma diagnostic and follow-up is its poor longitudinal repeatability, particularly in severe glaucoma [35]. The present results however are more align with observations by Lee et al. [36], which point to a diagnostic advantage of OCT compared to SLP. In paediatric populations, OCT was shown to provide a reliable longitudinal reproducibility [17, 18] combined with a higher image quality compared to SLP (measured by their respective software) [37]. Also, the use of OCT to analyse the macular ganglion cell complex represents an important diagnostic addition for glaucoma in adults [24] and in children [38, 39]. Finally, recent commercialisation of hand-held OCT devices opened new opportunities for paediatric glaucoma diagnostics by allowing the examination of patients too small for conventional OCT devices (i.e. not reaching the chin rest) or in a supine position, e.g., under general anaesthesia [5]. Similarly to reports in adults though [13], the moderate sensitivity of OCT measurements of the pRNFL make it unsuitable for usage as a standalone screening or diagnostic method in childhood glaucoma. In summary, due to the fact that most diagnostically reliable SLP parameters are unavailable in children, OCT seems the more accurate method for pRNFL thickness measurements in paediatric glaucoma.

Facing the difficult task of diagnosing glaucoma in children, objective morphologic diagnostic methods are of high clinical value. The presented data show a diagnostic superiority of pRNFL thickness measurement using OCT compared to SLP. Along with clinical parameters like IOP, ONH morphology, and functional tests, the additional role of pRNFL thickness measurements in paediatric glaucoma should be validated in broader, prospective studies.

## Data Availability

Original data can be provided upon request.
